# Chronic Activation of the G Protein-Coupled Receptor 30 with Agonist G-1 Attenuates Heart Failure

**DOI:** 10.1371/journal.pone.0048185

**Published:** 2012-10-26

**Authors:** Shoulei Kang, Ying Liu, Di Sun, Chunle Zhou, Aiying Liu, Chuanying Xu, Yanling Hao, Dongye Li, Changdong Yan, Hong Sun

**Affiliations:** 1 Department of Physiology, Xuzhou Medical College, Xuzhou, 221002, China; 2 Institute of Cardiovascular Disease Research, Xuzhou Medical College Affiliated Hospital, Xuzhou, 221002, China; Temple University, United States of America

## Abstract

G protein-coupled receptor (GPR) 30 is a novel estrogen receptor. Recent studies suggest that activation of the GPR30 confers rapid cardioprotection in isolated rat heart. It is unknown whether chronic activation of GPR30 is beneficial or not for heart failure. In this study we investigated the cardiac effect of sustained activation or inhibition of GPR30. Female Sprague–Dawley rats were divided into 7 groups #2Q1: sham surgery (Sham), bilateral ovariectomy (OVX), OVX+estrogen (E_2_), OVX+isoproterenol (ISO), OVX+ISO+G-1, OVX+ISO+E_2_+G15, OVX+ISO+E_2_. ISO (85 mg/kg×17 day, sc) was given to make the heart failure models. G-1(120 µg/kg·d×14 day) was used to activate GPR30 and G15 (190 µg/kg·d×14 day) was used to inhibit GPR30. Concentration of brain natriuretic peptide in serum, masson staining in isolated heart, contractile function and the expression of β_1_ and β_2_- adrenergic receptor (AR) of ventricular myocytes were also determined. Our data showed that ISO treatment led to heart failure in OVX rats. G-1 or E_2_ treatment decreased concentration of brain natriuretic peptide, reduced cardiac fibrosis, and enhanced contraction of the heart. Combined treatment with β_1_ (CGP20712A) and β_2_-AR (ICI118551) antagonist abolished the improvement of myocardial function induced by G-1. We also found that chronic treatment with G-1 normalized the expression of β_1_-AR and increased the expression of β_2_-AR. Our results indicate that chronic activation of the GPR30 with its agonist G-1 attenuates heart failure by normalizing the expression of β_1_-AR and increasing the expression of β_2_-AR.

## Introduction

Heart failure (HF) is a complex clinical syndrome that can result from any structural or functional cardiac disorder, it impairs the ability of the ventricle to fill with or eject blood. Despite significant advances in understanding the mechanisms underlying this disease, current treatments for HF have not been satisfied. It is recognized that sympathetic nervous system is one of the most important mechanisms regulating cardiac function, mainly through activation of β-AR [1]. Catecholamine such as epinephrine and norepinephrine are agonists of adrenoceptor in vivo, and levels of circulating catecholamine increased in patients with heart failure [2]. The development of heart failure also associated with diminishment of β-AR responsiveness [3], which assumed that reduced the density of β_1_-AR, but β_2_-AR was unaffected [4], [5]. Blockade of β_1_ and desensitization of β_2_-AR could reduce cardiac fibrosis which induced by ISO [6]. We and others have shown that overexpression of β_2_-AR protected the hearts against ischemia/reperfusion (I/R) or chronic hypoxia injury [7], [8], and played a beneficial role in heart failure [9].

Pre-menopausal women have reduced risk for cardiovascular disease, and the incidence of cardiovascular disease increased after menopause. Studies on animal models have also suggested that estrogen played an important role in cardioprotection [10]. There are three different forms of the estrogen receptor, usually referred to as α (ERα), β (ERβ), and the third G protein-coupled estrogen receptor (GPER), here referred as GPR30. Previous study showed that GPR30 subcellular localized in the endoplasmic reticulum and plasma membrane [11], [23], [24], and expressed in a variety of tissues such as heart, vascular, liver and ovarian in human and rats [16], [25]. Estrogen binds to the ERs, on the one hand translocates to the nucleus to produce genomic actions; on the other hand, confers rapid non-genomic actions [12]. Anne M. and his colleagues have reported that GPR30 specific agonist G-1 reduced post-ischemic dysfunction and infarct size after I/R, they found that the protection was blocked by the addition of the PI3K inhibitor [13]. Others have also found the similar results [14]–[16].

In addition to the rapid effects caused by activation of GPR30, its chronic effects have also been identified. It was reported that genetic deletion of GPR30 was associated with visceral adiposity in both male and female animals [17]. Jewell A. Jessup and his colleagues have shown that chronic GPR30 activation attenuated changes in left ventricular remodeling due to prolonged intake of a high salt diet [18]. We have reported oestrogen conferred cardioprotection by changing the expression of β_1_- and β_2_-AR [7], however oestrogen can bind to classical estrogen receptor and the novel estrogen receptor GPR30, whether separate activation of GPR30 with G-1 is beneficial for ISO induced heart failure, or changes the expression of β-AR has not been reported.

## Results

### General Features of Experimental Animals

Serum estrogen levels, uterine weight decreased and body weight increased significantly after the ovaries were removed. There were no significant differences between each group in body length. Compared with the Sham or OVX+E_2_ group, OVX treatment increased heart weight, but it was not significant. ISO plus OVX increased heart weight and heart weight/body length ratio compared with OVX group. G-1 or E_2_ but not E_2_+G15 could eliminate the increasing of the heart weight caused by OVX plus ISO. E_2_ and E_2_+G15 but not G-1 could increase uterine weight (table 1).

**Table 1:** General features.

	Sham	OVX	OVX+ISO	OVX+ISO+G1	OVX+ISO+E_2_+G15	OVX+ISO+E_2_
Number of rats	10	10	10	10	10	10
Body weight (g)	243±7	350±10*	345±10*	360±9*	353±7*	252±8#
Body length (mm)	203±7	210±5	195±10	198±7	205±4	212±8
Heart weight (mg)	850±23	910±19	1210±14*$	1007±25#	1290±23	950±22#
Heart weight/body length ratio (mg/mm)	4.25±0.13	4.31±0.11	6.50±0.16*$	5.12±0.12#	6.13±0.18	4.40±0.08#
Uterine weight (mg)	650±90	120±30*	160±51*	119±20*	623±31*	662±28#
Serum estradiol(pg/ml)	65.18±8.11	12.15±1.36*	10.87±0.79*	13.41±1.62*	62.54±1.06*	63.27±10.20#

Each value represents the mean±S.E.M. n = 10,

* P<0.05 versus Sham

# P<0.05 versus OVX+ISO and $p<0.05 versus OVX group.

### G-1 Treatment Increased the Ratio of Phosphorylated AKT

**Figure 1:**
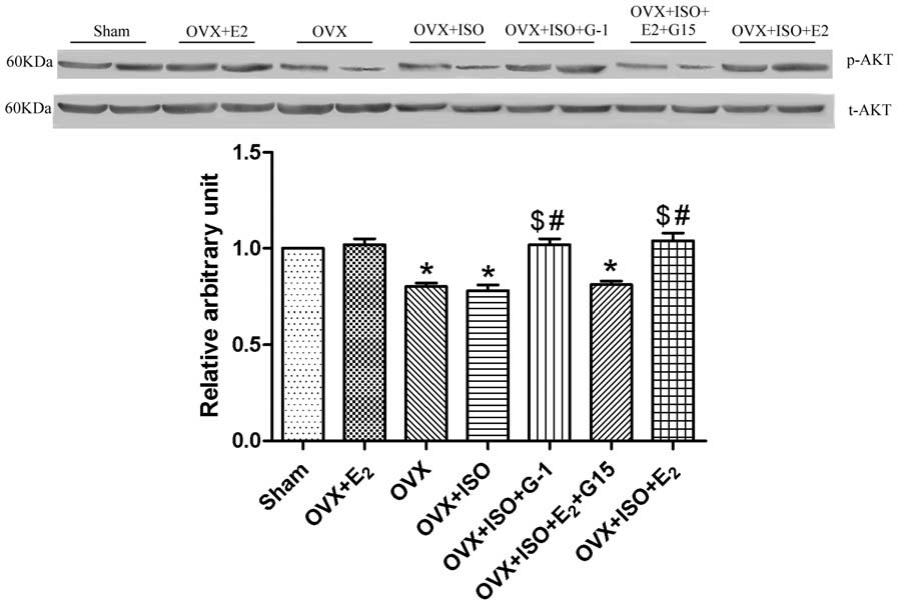
Expression of p-AKT. The relative arbitrary unit for sham group was assigned as figure (A). Figure (B) showed the expression of p-AKT. Each value represents the mean±S.E.M. n = 10 hearts in each group, *P<0.05 versus Sham, #P*<*0.05 versus OVX+ISO, $p<0.05 versus OVX.

In the experiment, OVX and OVX + ISO group have lower ratio of phosphorylated AKT than the Sham or OVX+E_2_ group, OVX + ISO +G-1 and OVX +ISO + E_2_ have higher ratio of phosphorylated AKT than OVX + ISO group, there was no significant differences between OVX +ISO + E_2_+G15 and OVX +ISO group (figure 1).

### G-1 Treatment Decreased BNP Levels in Heart Failure Model

**Figure 2:**
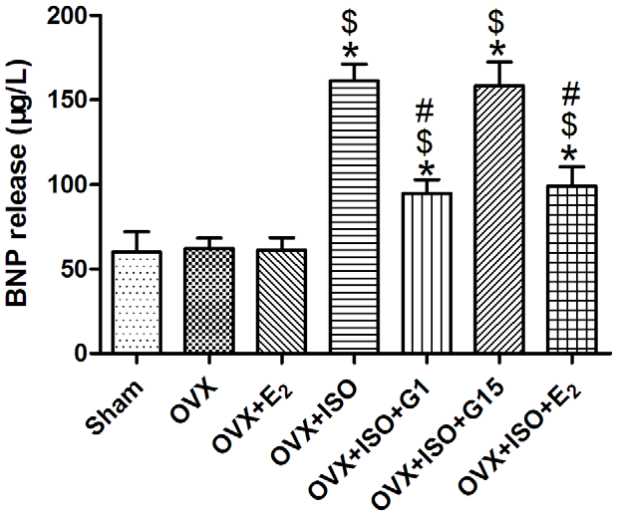
BNP activity of each group in serum. Each value represents the mean±S.E.M.; n = 10 hearts in each group. *P<0.05 versus Sham, #P<0.05 versus OVX+ISO, $p<0.05 versus OVX.

We assayed the concentration of brain natriuretic peptide (BNP) (in µg/L) in plasma before the animals were sacrificed. Ovariectomy induced a slight increase in the release of BNP compared with the Sham or OVX+E_2_ group. After ISO treatment, BNP release increased in all groups, but the OVX+ISO+G1/E_2_ group had a lower BNP release than OVX+ISO group (figure 2).

### G-1 Treatment Improved Cardiac Function in Heart Failure Model

Hearts were equilibrated for 30 minutes with KHB (Krebs–Henseleit buffer), and then we recorded the cardiac function data of each group. We found that there were no significant differences before ISO treatment among all groups. ISO treatment decreased LVDP, ±dp/dt and RPP, and increased LVEDP significantly. Administration of G1 or E_2_ increased LVDP, ±dp/dt, RPP, and decreased LVEDP, however G15 treatment can not cause such changes (table 2).

**Table 2:** Cardiac function of each group.

	LVDP mmHg	LVEDP mmHg	+dp/dt mmHg/s	-dp/dt mmHg/s	HR beats/min	RPP mmHg/min
Sham	89.7±8.6	5.9±0.4	1896.5±156.2	1672.3±123.2	283.5±16.8	26358.9±116.8
OVX	82.6±7.5	5.8±0.7	1859.2±147.3	1536.7±115.6	281.2±17.1	23065.2±113.1
OVX+ISO	39.8±3.2*$	16.8±2.9*$	923.4±87.8*$	565.2±64.6*$	223.4±15.8*$	8697.3±43.4*$
OVX+ISO+G-1	47.8±3.6*#$	11.2±1.7*#$	1394.9±97.1*#$	1022.4±78.1*#$	241.2±18.2*$	11327.6±67.9*#$
OVX+ISO+E_2_+G15	38.3±2.7*$	17.5±3.1*$	932.0±77.3*$	523.1±58.3*$	213.2±19.1*$	8093.8±47.6*$
OVX+ISO+E_2_	50.1±3.4*#$	10.8±2.2*#$	1411.3±106.3*#$	1103.4±88.2*#$	234.2±18.3*$	11706.3±74.1*#$

Each value represents the mean±S.E.M. n = 10,

* P<0.05 versus Sham.

# P<0.05 versus OVX+ISO, and $p<0.05 versus OVX group.

### G-1 Treatment Decreased the Fibrosis of the Failing Heart

**Figure 3:**
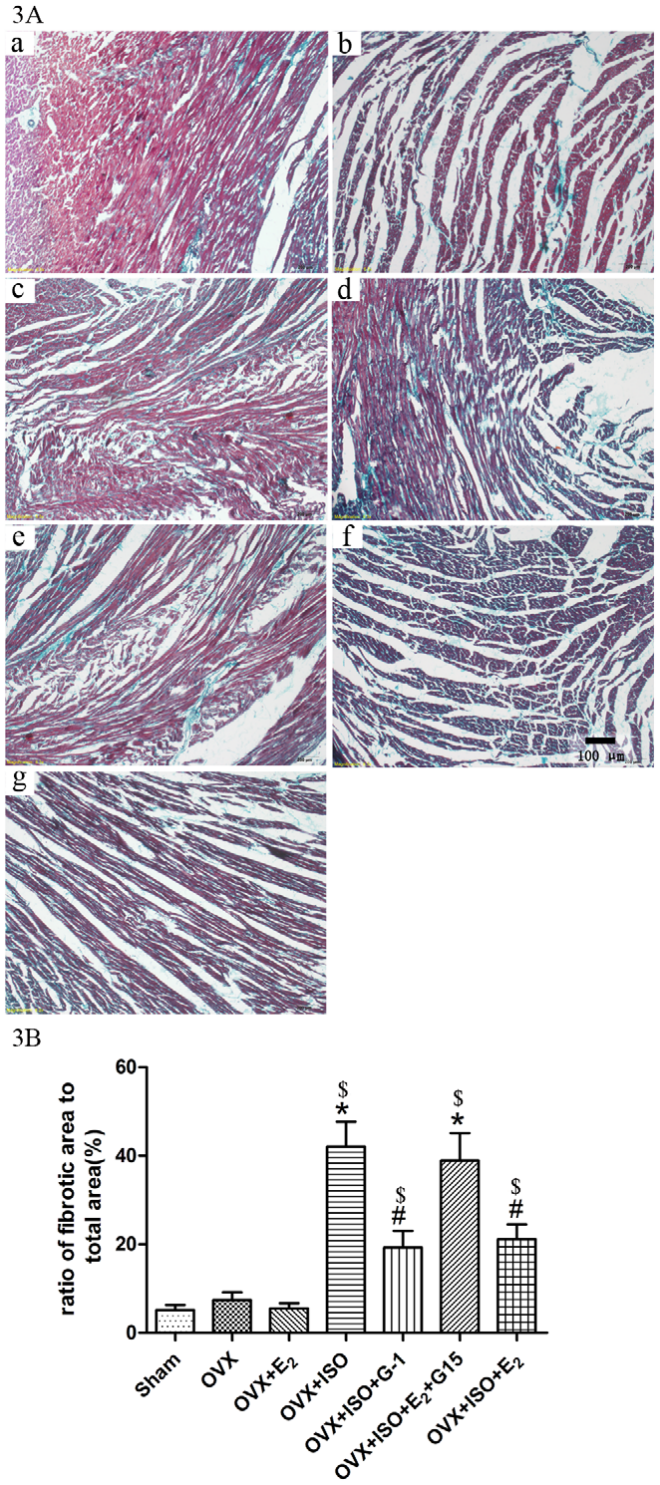
Masson staining of the heart. 3A: The fibrotic areas were stained green, and the normal cardiac myocytes were stained red and normal nuclei were stained blue. Representation of each group as following: a: Sham group; b: OVX group; c: OVX+ISO group; d: OVX+ISO+G-1 group; e: OVX+ISO+E_2_+G15 group; f: OVX+ISO+E_2_ group 3B: Each value represents the mean±S.E.M. n = 6, *P<0.05 versus Sham, #P<0.05 versus OVX+ISO, and $p<0.05 versus OVX group.

Masson staining showed that fibrotic areas were stained green, and the normal cardiac myocytes were stained red. The green areas were lower in Sham, OVX, OVX+E_2_ groups, stained sections showed increased fibrosis in OVX+ISO and OVX+ISO+E_2_+G15 group. OVX+ISO+G-1/E_2_ group has lower fibrotic areas than OVX+ISO group (figure 3).

### G-1 Treatment Enhanced Contraction of Myocardial Cells

**Figure 4:**
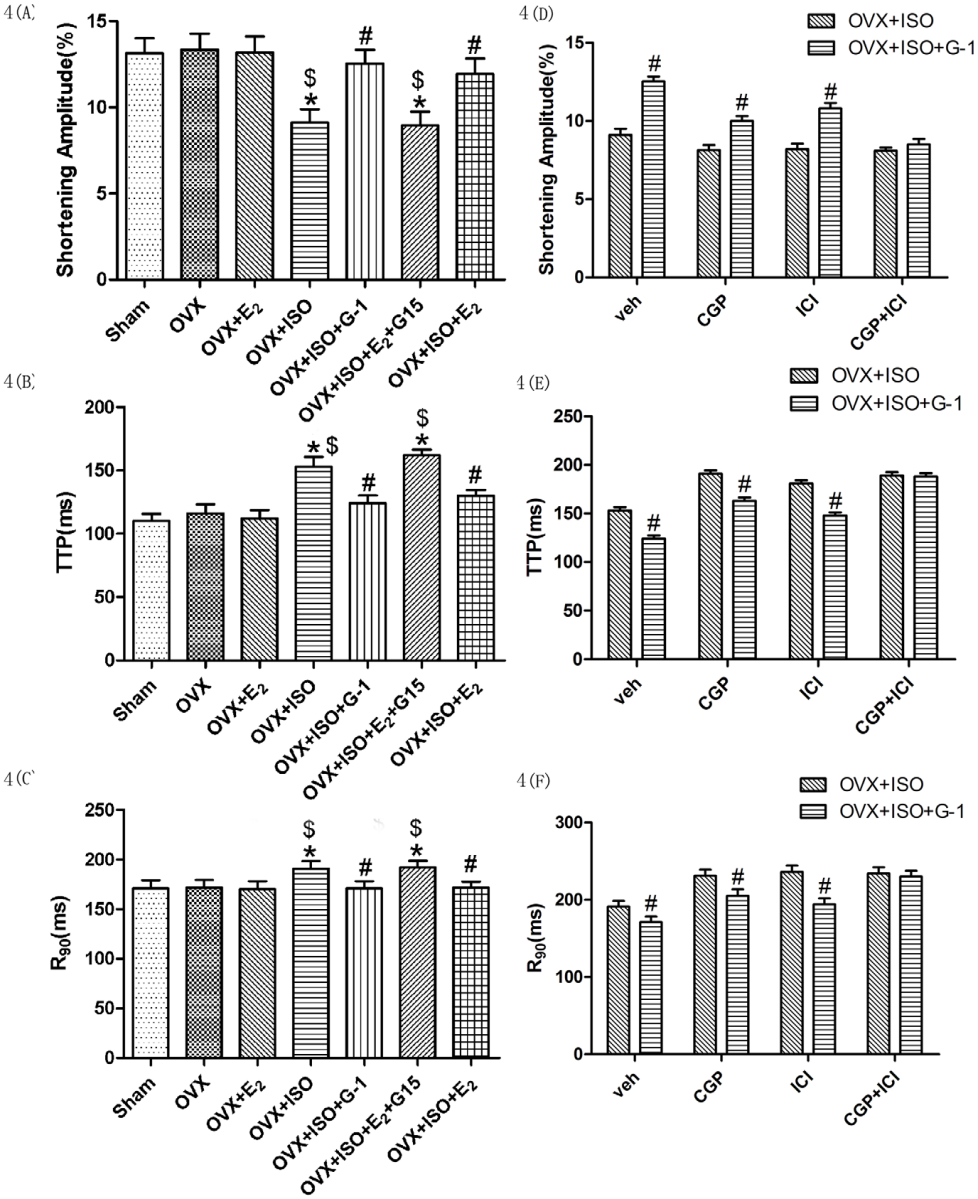
Function determination of the single cardiac cell. Myocytes of the six groups incubated in veh medium, shortening amplitude, TTP and R90 are shown as figure (A), (B) and (C) respectively. Data shown are mean± S.E.M. n = 10 hearts. *p<0.05 versus Sham, #p<0.05 versus OVX+ISO and $p<0.05 versus OVX. OVX+ISO and OVX+ISO+G-1 groups were incubated in veh, CGP or ICI medium. Shortening amplitude, TTP and R90 are shown as figure (D), (E) and (F) respectively. Data shown are mean±S.E.M. n = 10 hearts. #p<0.05 versus OVX+ISO of each subgroup.

First we observed contractility of myocytes isolated from Sham, OVX, OVX+ E_2_, OVX+ISO, OVX+ISO+G-1, OVX+ISO+E_2_+G15 and OVX+ISO+E_2_ which incubated with vehicle medium. We found that myocardial function decreased in OVX+ISO group compared with OVX group, which reflected in decreasing of contraction amplitude, and extending of systolic and diastolic. G-1 or E_2_ improved myocardial function, but E_2_+G15 had no effect on the improvement (figure 4A, 4B and 4C). This indicated that the protection was induced by activation of G protein-coupled receptor. To further study the relationship between the protective effect of G-1 and β-AR, we incubated myocytes isolated from OVX+ISO and OVX+ISO+G-1 with CGP, ICI or CGP+ICI. And then we found that, there were significant differences between OVX+ISO and OVX+ISO+G-1 in CGP or ICI medium, but in CGP+ICI medium, we didn’t find the difference (figure 4D, 4E, 4F). These indicated that the protection of G-1 may associate with both β_1_ and β_2_ -AR.

### β1- and β2-AR Protein Expressions in Ventricular Myocytes

**Figure 5:**
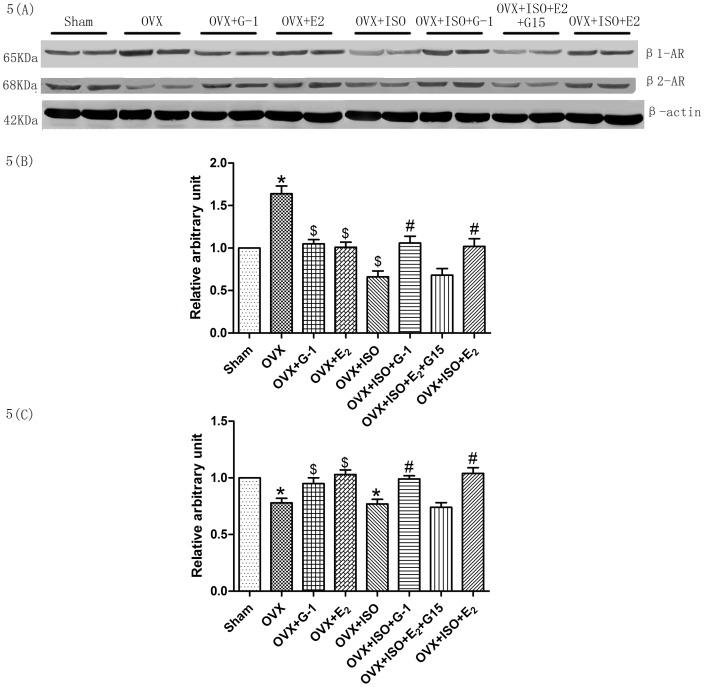
The expression of beta-AR. The expression of β-actin was detected as an internal standard. The relative arbitrary unit for sham group was assigned as (A). Figure (B) and (C) showed the expression of β_1_-AR and β_2_-AR. Each value represents the mean±S.E.M. n = 10 hearts in each group, *P<0.05 versus Sham, #P*<*0.05 versus OVX+ISO, $p<0.05 versus OVX.

Our research showed that the protective effect of G-1 in heart failure can be abolished by CGP+ICI, which indicated that the protective effect of G-1 may be relevant to β-AR, so we determined whether the expression of β_1_- or β_2_-AR was changed by G-1 treatment. We added two groups OVX+G-1 and OVX+E_2_ for ovariectomy could change the expression of β-AR [7]. We found that compared with Sham group, the expression of β_1_-AR increased in OVX group, G-1 or E_2_ treatment decreased the expression in OVX group. However OVX plus ISO treatment could also decreased the expression of β_1_-AR compared with OVX group, that may because sustained treatment with isoproterenol diminished β-AR responsiveness, the expression of β_1_-AR decreased is one of the manifestations [4]. OVX+ISO+G-1/E_2_ treatment increased the expression of β_1_-AR compared with OVX+ISO group. All these above indicated that G-1/E_2_ normalized the expression of β_1_-AR: decreasing the expression in OVX group and increasing the expression in OVX+ISO group. We also found that the expression of β_2_-AR decreased after ovariectomy no matter with ISO treatment or not, G-1 or E_2_ treatment increased the protein expression of β_2_-AR (figure 5).

## Discussion

In this study, we found that chronic treatment with GPR30 agonist G-1 attenuated heart failure in female SD rats. As a novel estrogen receptor GPR30 plays an important role in cardiac protection and gets more and more attention recently. In the paper, ISO treatment was used in ovariectomized rats to prepare for heart failure model [19]. BNP concentration in serum, hemodynamic, Masson’s trichrome staining in isolated heart, contractile function of ventricular myocytes were determined in the study, we also determined the effects of β_1_- and β_2_-AR antagonist on the function of myocytes, western blot method was used to determine the protein expression of p-AKT, β_1_- and β_2_-AR. Our data indicate that chronic G-1 treatment attenuated heart failure which induced by ISO, and the protective effect may be associated with regulating the expression of β-AR.

In our study, continuously and chronic effect of G-1 was observed. GPR30, as one of the estrogen receptor, can be activated by E_2_, in order to prevent the interference of E_2_, rats were ovariectomized. Here ISO treatment was used to get a heart failure model as previous described [19]. As our data shown, in OVX group, with out the protection of E_2_ (exogenous), ISO treatment caused significantly damage to the heart compared with E_2_ treatment, which indicated that estrogen played an important role in protection of the heart. To study the protective effect of GPR30, G1, E_2_+G15 and E_2_ were administrated. The amount of drugs was determined following the method mentioned in Lindsey SH.’s article [21]. First of all, determine drug affinity for GPR30 in the article [20], [21], affinities as shown below: G-1 11 nM, G15 20 nM and E_2_ 5.7 nM; second determine the concentration of G-1 and G15 according to the concentration of E_2_ which our laboratory used before [7] and then determine the ratio of their affinities to GPR30, the amount of drugs was determined: G-1 120 µg/kg·d, G15 190 µg/kg·d, E_2_ 40 µg/kg·d.

We measured animals’ weight before they were killed, G-1 treatment didn’t change weight gain induced by ovariectomy, which was consistent with the results of Lindsey SH.’s research [21], and E_2_ or E_2_+G15 treatment decreased weight gain induced by ovariectomy which in line with our previous study [7], [31], [32], possibly because ERα and ERβ played a role in regulating body weight [21]. Other indications in our experiment showed that E_2_+ G15 treatment didn’t play cardiac protection roles which indicated that the chronic activation of GPR30 is responsible, and not ERα and ERβ.

PI3K-AKT pathway is the downstream pathway of GPR30, and G-1 treatment increased phosphorylation of AKT. In our experiment, we determined the phosphorylation of AKT and found that G-1 or E2 treatment increased the phosphorylation of AKT, G15+E_2_ treatment didn’t increased the phosphorylation of AKT. This indicated that the special agonist G-1 activated GPR30.

BNP is mainly present in the left and right atria, the physiologic actions of it are similar to ANP (atrial natriuretic peptide) and include decrease in systemic vascular resistance and central venous pressure as well as an increase in natriuresis. The level of its secretion is closely related to the changes of ventricular filling pressure, when heart failure occurred, ventricular filling pressure raised and the secretion of BNP increased. The increase of the secretion was positively correlated to the degree of heart failure. So the concentration of BNP in serum could be an indicator to assess the severity of heart failure.

In the experiment, the concentration of BNP in OVX+ISO group increased significantly compared with OVX group, this is in according with the hemodynamics resulst. In OVX+ISO+G-1 group, the concentration of BNP decreased compared with OVX+ISO group, this indicated that G-1 treatment conferred cardiac protective effect in ISO induced heart failure model.

We have detected hemodynamic in organ levels, found that ISO treatment diminished cardiac ejection and G-1 treatment enhanced the ability of the cardiac ejection, this indicated that G-1 conferred cardiac protective effect. As G-1 could reduce vascular tone and dilate rodent arterial blood vessels [17], and β-AR antagonist also had the role of the vasodilator, in order to exclude the impact of these roles, we isolated cardiac myocytes with collagen digest method and detected systolic and diastolic function in single cells. In this way, we conclude that G-1, at least could act directly on myocardial cells in the protective effect of the failing heart.

We isolated cardiac myocytes of OVX+ISO and OVX+ISO+G-1 group, cultured with β_1_-AR antagonist CGP20712A, β_2_-AR antagonit ICI118551, we found that treatment with CGP or ICI separately could not abolish the improvement of the cell contraction., but combination treatment with CGP and ICI could abolish the improvement completely. This indicated that the protective of G-1 may associate with both β_1_-AR and β_2_-AR.

#3Q3Although there is a group with antagonist group, the ligand specificity in vivo is still limitation in vivo study, for example the antagonist drugs may reach to the liver, brain or other organs, which confer the systolic changes of the bodies.

The sympathetic nervous system is critically involved in the regulation of cardiac function through β-AR. Activation of β_1_-AR results in augmentation of cardiac activity (positive inotropic effect), including an increase in heart rate and atria-ventricle conduction velocity and enhancement of myocardial contraction [33]. Roth DM has pointed that overexpression of β_1_ receptors caused cardiac damage [25]. Our laboratory has found that the expression of β_1_-AR increased in ovariectomized female rats compared with the Sham group [7], which indicated that estrogen may play an important role in regulate the expression of β_1_-AR thus conferred cardiac protection effect. In this paper, we found that the expression of β_1_-AR increased in OVX group, G-1 or E_2_ treatment decreased it, and we didn’t observed cardiac damage indications in OVX group, here we speculated ovariectomized is just a risk factor for hearts. However ISO treatment decreased the expression of β_1_-AR and produced injury effect which may be attributed to continuous stimulation of catecholamine led to decline in receptor number and reduce of the function [4], G-1 or E_2_ treatment could reduce the injury and increased the expression of β_1_-AR compared with OVX+ISO group. Taken together, G-1 or E_2_ treatment regulated protein β_1_-AR in the protective effects.

Unlike β_1_AR, activation of β_2_-AR plays a beneficial role in hearts. Sustained β_1_-AR stimulation promotes apoptotic death of cardiomyocytes, sustained stimulation of β_2_-AR protects myocytes against a wide range of apoptotic insults [27]. Similarly, some studies showed that overexpression of β_2_-AR conferred cardiac protective effect in the heart [8], [28] which was consistent with our results.

In our opinion, treatment with the estrogen hormone agonist G-1 could increase the expression of β_2_-AR. Interestingly, other hormones or models could also regulate the expression of β_2_-AR in the body. For instance, Penna C has reported sub-chronic nandrolone pretreatment increased the expression of β_2_-AR [28], thyroid hormones increased the mRNA of β_2_-AR in heart [29], and in diabetic heart model, the expression of β_2_-AR decreased [30]. However whether the mechanism of protective effects of G-1 which changed the expression of β_2_-AR is direct or indirect effects such as regulating the secretion of other hormones is unknown, the mechanisms remain to be further studied.

Taken all together, in this study we found that chronic treatment with G-1 attenuated heart failure by increased the expression of β_2_-AR and normalized the expression of β_1_-AR in ovariectomized rats. This is the first time we have reported chronic treatment with G-1 is beneficial for the heart failure.

## Materials and Methods

### Animals and Reagents

Female Sprague-Dawley (SD) rats weighing 180–220 g (200±20 g) were obtained from the Experimental Animal Centre of Xuzhou Medical College and all studies were approved by the Animal Ethics Committee of the Medical College of Xuzhou (permit number: xz11-12540) and conform to the Guide for the Care and Use of Laboratory Animals published by the US National Institutes of health (NIH publication No. 85-23, revised 1996). The main reagents used in the experiments are as follows: isoproterenol hydrochloride (Sigma-Aldrich, St.Louis, MO, USA), G-1 (EMD Chemicals, San Diego, CA, USA), G15(EMD Chemicals, San Diego, CA, USA), 17β-estradlol (ABCR, Germany), ICI118551 (tocris, Bristol, UK), CGP20712A (Sigma-Aldrich, St.Louis, MO, USA), wortmannin (Merck, Darmstadt, Germany), in situ cell death detection kit, pod (Roche, Indianapolis, IN,USA), rabbit polyclonal anti β_1_-AR, β_2_-AR, p-AKT, t-AKT (Santa Cruz, CA, USA), brain natriuretic peptide (BNP) ELISA Kits (Fengxiang Bio-technology, Shanghai, China), Masson’s Trichrome Stain Kit (Yuanye Bio-technology, Shanghai China).

The animals were anesthetized with sodium pentobarbitone (60 mg/kg, I.P.), corneal reflex disappeared, sensation of pain reduced and muscle tension decreased was the sign of the success of anesthesia. Incision was chosen at the side of the spine, separated the muscle layer and opened the peritoneum, then the ovaries were exteriorized, ligated and removed in OVX group. In Sham group, visualization of the ovaries through incisions into the abdominal cavity and then closure of the wounds. All surgeries were carried out under sterile conditions. ISO (85 mg/kg, sc) treatment was continued for 17 d to get the heart failure model [19]. Osmotic minipump (model 2006, Alza Corp., Palo Alto, CA, USA) implanted s.c. at the dorsum of the neck. In the surgery, pentobarbitone (60 mg/kg, I.P.) was used as an anaesthetic, corneal reflex disappeared, sensation of pain reduced and muscle tension decreased was the sign of the success of anesthesia. G-1, E_2_+G15 or E_2_ was pumped via the minipump at the dose of 120 µg/kg·d, 190 µg/kg·d, 40 µg/kg·d respectively, treatment lasted for 2 weeks. The dose of G-1, G15, E_2_ based on the affinity to GPR30 [20], [21] and our previous studies [7]. In general, seven groups were divided: Sham, OVX, OVX+E_2_, OVX+ISO, OVX+ISO+G-1, OVX+ISO+E_2_+G15, OVX+ISO+E_2_.

### Measurement of BNP Concentration in Plasma

We collected the blood from tail vein of the rat before it was killed, added an appropriate amount of heparin and then centrifuged at the rate of 2500 rpm for 15 min. The concentration of BNP was assayed as the instructions of ELISA kits. All samples were assayed in triplicate.

### Isolation of the Heart and Heart Perfusion

Intraperitoneal injection with 5000 U/kg heparin was implemented, 15 minutes later, 150 mg/kg pentobarbital was injected for anesthesia, corneal reflex disappeared, sensation of pain reduced and muscle tension decreased was the sign of the success of anesthesia. A transverse incision was made, the abdominal cavity was exposed, hearts were quickly isolated and rinsed with ice-cold Ca^2+^ free buffer as we have reported before [7]. Oxygenated KHB was perfused through Mouse heart perfusion system (TME Technology, Chengdu, CHINA) in the experiment [22].

### Determination of Cardiac Function

Linked aortic side of the isolated heart to perfusion device, left ventricular pressure was recorded using a Biopac system (BIOPAC) via a pressure sensor Millar transducer instrument (1.4F, Millar). Hearts were equilibrated for 30 minutes with KHB. Heart rate (HR), left ventricular systolic pressure (LVSP), left ventricular enddiastolic pressure (LVEDP), and the rate in rise and fall of ventricular pressure (+dp/dtmax, -dp/dtmax) were recorded as left ventricular functional parameters. The left ventricular developed pressure (LVDP) were calculated as LVDP = LVSP-LVEDP; rate pressure product (RPP) was calculated as RPP = HR×LVDP.

### Masson’s Trichrome Stain

Rat hearts were perfused with KHB for 20 minutes to remove the residual blood, and then fixed with 10% formalin before embedding in paraffin. All hearts were embedded in a cross section orientation, and all slices were cross sections of the heart. All slices were taken from the midpoint of the left ventricles. Four-micrometer slices were deparaffinized and rehydrated. Then the instruction of masson’s trichrome stain kit was followed. Microscope (IX 71, OLYMPUS, Japan) was used to get the pictures. The normal cardiac myocytes were stained red, and fibrotic areas were stained green.

### Isolation and Culture of Myocardial Cells

Ventricular myocytes were isolated from the hearts as we described before [7], [22]. Cardiac ventricular myocytes were isolated from the hearts then the cells were suspended in dulbecco’s minimal essential medium (DMEM) at a density of 3×10^5^ cells per well, finally took them to carbon dioxide incubator (Heraeus, Germany), parameters were set as below: 5% CO_2_, 37°C, cultured for 1 hour to stabilize the cells. The rat cardiomyocytes were incubated with vehicle (veh), CGP20712A (CGP, 300 nM), or ICI118551 (ICI, 55 nM), for 2 h to study the impact of different β-AR antagonists on shortening amplitude in rat myocytes.

### Myocyte Contractility

After myocardial cells were isolated, each group was divided into three subgroups: veh, CGP, ICI. A few drops of solution containing ventricular myocytes were added to an open chamber on the stage of an inverted microscope (Olympus, Japan). After 5 min, to allow cells to spontaneously attach to the floor of the chamber, they were superfused at 2 ml/min with KHB (containing 2.0 mM Ca2+ and 100 nM ISO) adjusted to pH 7.4 by equilibration with 95% O2–5% CO2. The ventricular myocytes were paced with an electrical field stimulation (0.5 Hz). Myocytes used were rod shaped with clear sarcomeres, and 10 consecutive contractions were averaged. The whole process was recorded by a video recorder (Panasonic, Japan) connect to the microscope. Then the video was sent to a computer, cut into pictures by the software VirtualDub, the process of contraction and relaxation was recorded and transformed into digital data by the software OpticalMeasure (presented by China’s National Defense University of Science and Technology). Contractile function was assessed using the following indices: shortening amplitude, the amplitude myocytes shortened upon electrical stimulation, an indicative of peak ventricular; contractility time-to-peak contraction (TTP), the duration of myocyte shortening, an indicative of systolic duration; and time-to-90% relaxation (R_90_), the duration to reach 90% relengthening, an indicative of diastolic duration.

### Western Blot

Ventricular myocytes were frozen in liquid nitrogen for analysis of p-AKT, β_1_- and β_2_-AR protein by western blotting. Myocytes were homogenized in ice-cold homogenization. We followed our previous method in this part [7]. The membranes were scanned into the computer, and relative intensity of bands was analysed by the software photoshop (Adobe, San Jose, CA, USA).

### Statistical Analysis

In each experimental series, data are presented as means±S.E.M. Statistical analysis was performed with GraphPad Prism 5.01 (GraphPad Software, San Diego, CA, USA). The contraction of the myocytes were analysed by two-way ANOVA, other data were analysed by one-way ANOVA followed by Bonferroni post hoc tests.

## References

[1] Xiao RP, Zhu W, Zheng M, Chakir K, Bond R, et al (2004) Subtype-specific beta-adrenoceptor signaling pathways in the heart and their potential clinical implications. Trends Pharmacol Sci. 25: 358–365. 10.1016/j.tips.2004.05.007 15219978

[2] Zhang GX, Kimura S, Nishiyama A, Shokoji T, Rahman M, et al (2005) Cardiac oxidative stress in acute and chronic isoproterenol-infused rats. Cardiovasc Res. 65: 230–238. 10.1016/j.cardiores.2004.08.013 15621051

[3] Hamdani N, de Waard M, Messer AE, et al (2008) Myofilament dysfunction in cardiac disease from mice to men. J Muscle Res Cell Motil. 29: 189–201. 10.1007/s10974-008-9160-y 19140019

[4] Bristow MR, Ginsburg R, Umans V, Fowler M, Minobe W, et al (1986) Beta 1- and beta 2-adrenergic-receptor subpopulations in nonfailing and failing human ventricular myocardium: coupling of both receptor subtypes to muscle contraction and selective beta 1-receptor down-regulation in heart failure. Circ Res. 59: 297–309. 10.1161/01.res.59.3.297 2876788

[5] Shizukuda Y, Buttrick PM (2002) Subtype specific roles of beta-adrenergic receptors in apoptosis of adult rat ventricular myocytes. J Mol Cell Cardiol. 34: 823–831. 10.1006/jmcc.2002.2020 12099721

[6] Brouri F, Hanoun N, Mediani O, Saurini F, Hamon M, et al (2004) Blockade of beta 1- and desensitization of beta 2-adrenoceptors reduce isoprenaline-induced cardiac fibrosis. Eur J Pharmacol. 485: 227–234. 10.1016/j.ejphar.2003.11.063 14757145

[7] Wu Q, Zhao Z, Sun H, Hao YL, Yan CD, et al (2008) Oestrogen changed cardiomyocyte contraction and beta-adrenoceptor expression in rat hearts subjected to ischaemia-reperfusion. Exp Physiol. 93: 1034–1043. 10.1113/expphysiol.2007.041939 18469068

[8] Dong H, Chen Q, Sun S, Yu H, Zhang Z (2010) Overexpression of beta(2)AR improves contractile function and cellular survival in rabbit cardiomyocytes under chronic hypoxia. Biochem Biophys Res Commun. 398: 383–388. 10.1016/j.bbrc.2010.06.076 20599724

[9] Sun J, Fu L, Tang X, Han Y, Ma D, et al (2011) Testosterone modulation of cardiac β-adrenergic signals in a rat model of heart failure. Gen Comp Endocrinol. 172: 518–525. 10.1016/j.ygcen.2011.04.019 21549119

[10] Murphy E, Steenbergen C (2007) Gender-based differences in mechanisms of protection in myocardial ischemia-reperfusion injury. Cardiovasc Res. 75: 478–486. 10.1016/j.cardiores.2007.03.025 17466956

[11] Revankar CM, Mitchell HD, Field AS, et al (2007) Synthetic estrogen derivatives demonstrate the functionality of intracellular GPR30. ACS Chem Biol. 2: 536–544. 10.1021/cb700072n 17655271

[12] Prossnitz ER, Arterburn JB, Smith HO, Oprea TI, Sklar LA, et al (2008) Estrogen signaling through the transmembrane G protein-coupled receptor GPR30. Annu Rev Physiol. 70: 165–190. 10.1146/annurev.physiol.70.113006.100518 18271749

[13] Deschamps AM, Murphy E (2009) Activation of a novel estrogen receptor, GPER, is cardioprotective in male and female rats. Am J Physiol Heart Circ Physiol. 297: H1806–H1813. 10.1152/ajpheart.00283.2009 PMC2781389 19717735

[14] Weil BR, Manukyan MC, Herrmann JL, Wang Y, Abarbanell AM, et al (2010) Signaling via GPR30 protects the myocardium from ischemia/reperfusion injury. Surgery. 148: 436–443. 10.1016/j.surg.2010.03.011 20434187

[15] Bopassa JC, Eghbali M, Toro L, Stefani E (2010) A novel estrogen receptor GPER inhibits mitochondria permeability transition pore opening and protects the heart against ischemia-reperfusion injury. Am J Physiol Heart Circ Physiol. 298: H16–H23. 10.1152/ajpheart.00588.2009 PMC2806134 19880667

[16] Patel VH, Chen J, Ramanjaneya M, Karteris E, Zachariades E, et al (2010) G-protein coupled estrogen receptor 1 expression in rat and human heart: Protective role during ischaemic stress. Int J Mol Med. 26: 193–199. 10.3892/ijmm_00000452 20596598

[17] Haas E, Bhattacharya I, Brailoiu E, et al (2009) Regulatory role of G protein-coupled estrogen receptor for vascular function and obesity. Circ Res. 104: 288–291. 10.1161/CIRCRESAHA.108.190892 PMC2782532 19179659

[18] Jessup JA, Lindsey SH, Wang H, Chappell MC, Groban L (2010) Attenuation of salt-induced cardiac remodeling and diastolic dysfunction by the GPER agonist G-1 in female mRen2.Lewis rats. PLoS One. 5: e15433. 10.1371/journal.pone.0015433 PMC2972725 21082029

[19] Parveen A, Babbar R, Agarwal S, Kotwani A, Fahim M (2011) Terminalia arjuna Enhances Baroreflex Sensitivity and Myocardial Function in Isoproterenol-Induced Chronic Heart Failure Rats. J Cardiovasc Pharmacol Ther. [Epub ahead of print] DOI: 10.1177/1074248411416816. 10.1177/1074248411416816 21828283

[20] Dennis MK, Burai R, Ramesh C, et al (2009) In vivo effects of a GPR30 antagonist. Nat Chem Biol. 5: 421–427. 10.1038/nchembio.168 PMC2864230 19430488

[21] Lindsey SH, Cohen JA, Brosnihan KB, Gallagher PE, Chappell MC (2009) Chronic treatment with the G protein-coupled receptor 30 agonist G-1 decreases blood pressure in ovariectomized mRen2.Lewis rats. Endocrinology. 150: 3753–3758. 10.1210/en.2008-1664 PMC2717873 19372194

[22] Hao Y, Sun Y, Xu C, Jiang X, Sun H, et al (2009) Improvement of contractile function in isolated cardiomyocytes from ischemia-reperfusion rats by ginkgolide B pretreatment. J Cardiovasc Pharmacol. 54: 3–9. 10.1097/FJC.0b013e3181a91410 19487958

[23] Otto C, Rohde-Schulz B, Schwarz G, Fuchs I, Klewer M, et al (2008) G protein-coupled receptor 30 localizes to the endoplasmic reticulum and is not activated by estradiol. Endocrinology. 149: 4846–4856. 10.1210/en.2008-0269 18566127

[24] Filardo E, Quinn J, Pang Y, Graeber C, Shaw S, et al (2007) Activation of the novel estrogen receptor G protein-coupled receptor 30 (GPR30) at the plasma membrane. Endocrinology. 148: 3236–3245. 10.1210/en.2006-1605 17379646

[25] O’Dowd BF, Nguyen T, Marchese A, Cheng R, Lynch KR, et al (1998) Discovery of three novel G-protein-coupled receptor genes. Genomics. 47: 310–313. 10.1006/geno.1998.5095 9479505

[26] Roth DM, Gao MH, Lai NC, Drumm J, Dalton N, et al (1999) Cardiac-directed adenylyl cyclase expression improves heart function in murine cardiomyopathy. Circulation. 99: 3099–3102. 10.1161/01.cir.99.24.3099 10377071

[27] Zhu W, Zeng X, Zheng M, Xiao RP (2005) The enigma of beta2-adrenergic receptor Gi signaling in the heart: the good, the bad, and the ugly. Circ Res. 97: 507–509. 10.1161/01.RES.0000184615.56822.bd 16166560

[28] Penna C, Abbadessa G, Mancardi D, Tullio F, Piccione F, et al (2008) Synergistic effects against post-ischemic cardiac dysfunction by sub-chronic nandrolone pretreatment and postconditioning: role of beta2-adrenoceptor. J Physiol Pharmacol. 59: 645–659. 19212001

[29] Viguerie N, Millet L, Avizou S, Vidal H, Larrouy D, et al (2002) Regulation of human adipocyte gene expression by thyroid hormone. J Clin Endocrinol Metab. 87: 630–634. 10.1210/jcem.87.2.8200 11836296

[30] Dinçer UD, Bidasee KR, Güner S, Tay A, Ozçelikay AT, et al (2001) The effect of diabetes on expression of beta1-, beta2-, and beta3-adrenoreceptors in rat hearts. Diabetes. 50: 455–461. 10.2337/diabetes.50.2.455 11272160

[31] Xu C, Liu A, Sun H, Sun Y, Wang G, et al (2010) beta2-Adrenoceptor confers cardioprotection against hypoxia in isolated ventricular myocytes and the effects depend on estrogenic environment. J Recept Signal Transduct Res. 30: 255–261. 10.3109/10799893.2010.488242 20602544

[32] Liu AY, Gao LP, Kang SL, Liu Y, Xu CY, et al (2011) Testosterone enhances estradiol’s cardioprotection in ovariectomized rats. J Endocrinol. 212: 61–69. 10.1530/JOE-11-0181 21965546

[33] Guan Y, Gao L, Ma HJ, Li Q, Zhang H, et al (2010) Chronic intermittent hypobaric hypoxia decreases beta-adrenoceptor activity in right ventricular papillary muscle. Am J Physiol Heart Circ Physiol. 298: H1267–H1272. 10.1152/ajpheart.00410.2009 20097768

